# Pericytes in Tumor Vessels

**DOI:** 10.3390/cells15201867

**Published:** 2026-10-14

**Authors:** Domenico Ribatti, Roberto Tamma, Francesco Pezzella

**Affiliations:** 1 Department of Translational Biomedicine and Neuroscience, University of Bari Medical School, 70124 Bari, Italy; 2 Nuffield Division of Laboratory Science, Radcliffe Department of Medicine, University of Oxford, John Radcliffe Hospital, Oxford OX3 9DU, UK; francesco.pezzella@ndcls.ox.ac.uk

**Keywords:** angiogenesis, metastasis, pericyte, tumor growth

## Abstract

Pericytes are specialized mural cells that closely interact with endothelial cells to regulate vascular development, stability, and homeostasis through signaling pathways including PDGF-B/PDGFR-β, TGF-β, Angiopoietin-1/Tie2, and N-cadherin. In cancer, they display remarkable phenotypic and functional plasticity, becoming key regulators of tumor angiogenesis and the tumor microenvironment. Disrupted pericyte–endothelial interactions contribute to the formation of abnormal, highly permeable blood vessels, promoting hypoxia, tumor progression, immune evasion, and metastasis. Beyond their vascular role, pericytes actively communicate with tumor-associated macrophages, cancer-associated fibroblasts, and extracellular matrix components, thereby influencing epithelial-to-mesenchymal transition, pre-metastatic niche formation, and therapeutic resistance. Increasing evidence also suggests that pericytes undergo phenotypic transitions that further support tumor progression. Conversely, restoring pericyte function and promoting vessel normalization have emerged as promising strategies to improve vascular integrity, tissue oxygenation, drug delivery, and the efficacy of anti-angiogenic therapies and immunotherapy. Preclinical and early clinical studies indicate that targeting pathways regulating pericyte recruitment and function, particularly in combination with endothelial-directed therapies, may produce synergistic anti-tumor effects. This review highlights the multifaceted roles of pericytes in tumor vascular remodeling, immune regulation, metastasis, and their potential as therapeutic targets in cancer.

## 1. Introduction

Perivascular mural cells known as pericytes were initially identified in 1871 by Carl Joseph Eberth and subsequently described by Charles-Marie Benjamin Rouget in 1873, who coined the term “Rouget cells”. In 1923, Karl Wilhelm Zimmermann designated them as “pericytes” to highlight their anatomical position surrounding the microvasculature.

Within microvascular beds, pericytes are strategically positioned across pre-capillary, mid-capillary, and post-capillary segments. Mid-capillary pericytes possess prominent longitudinal processes extending along multiple endothelial cells, accompanied by secondary circumferential branches that partially encircle the capillary lumen. Conversely, pericytes at pre- and post-capillary levels display a more branched, stellate morphology. Tissues differ widely in their numbers of pericytes or their endothelial coverage (retina > lung > skeletal muscle > cardiac muscle > adrenal gland) [1]. In the central nervous system (CNS), pericytes represent a major structural element of the blood–brain barrier (BBB), yielding an exceptionally high pericyte-to-endothelial cell ratio. Single-cell RNA sequencing has revealed significant differences in gene expression among pericytes from different organs, indicating distinct functional role [2].

Although pericytes and vascular smooth muscle cells (VSMCs) both represent mural lineages, they occupy distinct microvascular domains and display discrete structural features. Pericytes reside within the microvascular basement membrane via extended cytoplasmic processes, whereas VSMCs assemble into multi-layered concentric coats surrounding larger arterial and venous vessels. A unique, universal biomarker for pericytes remains elusive; consequently, phenotypic identification relies on panel-based approaches tailored to specific organs, developmental stages, and pathological states. Commonly evaluated positive markers include platelet-derived growth factor receptor-beta (PDGFR-β), chondroitin sulfate proteoglycan 4 (CSPG4/NG2), CD13, desmin, alpha-smooth muscle actin (α-SMA), regulator of G-protein signaling 5 (RGS5), SUR2, Kir6.1, endosialin, and delta-like 1 homolog (DLK1) [3].

It is possible to discriminate between pericytes, vascular smooth cells, and fibroblasts utilizing different biomarkers. Pericytes are typically positive for PDGFRβ, NG2, CD13, and CD146. They are notably negative for Myh11 (mature smooth muscle myosin heavy chain) and standard endothelial or immune tags (CD31, CD34, CD45). Vascular smooth cells richly express mature contractile proteins like Myh11 (SMMHC) and TAGLN (SM22α). While they share PDGFRβ and NG2 expression with pericytes, they express profoundly higher baseline levels of α-SMA. Fibroblasts are characterized by structural matrix markers such as PDGFRα, CD34, Vimentin, Lumican (LUM), and Decorin (DCN).

The study of pericytes’ ontogeny and capacity for differentiation is complicated by the absence of a single marker for their identification. There is still much to learn how and when the origin of pericytes in an organ is determined and whether pericytes have the potential to differentiate in vivo. During vascular development, endothelial cells secrete PDGF-B to recruit mesenchymal precursors. Interaction between these precursor cells and endothelial cells triggers latent transforming growth factor beta 1 (TGFβ1) activation, driving mural lineage commitment, inducing pericyte marker expression, and restraining endothelial hyperproliferation [4,5]. TGF-β signaling coordinates the commitment of uncommitted progenitors into PDGFR-β-positive precursors, which subsequently migrate toward endothelial-derived PDGF-B gradients [6].

Genetic ablation of PDGF-B or PDGFRβ in murine models causes severe deficits in perivascular coverage, endothelial hyperplasia, microaneurysm formation, vascular instability, and severe edema [7].

**Figure 1:**
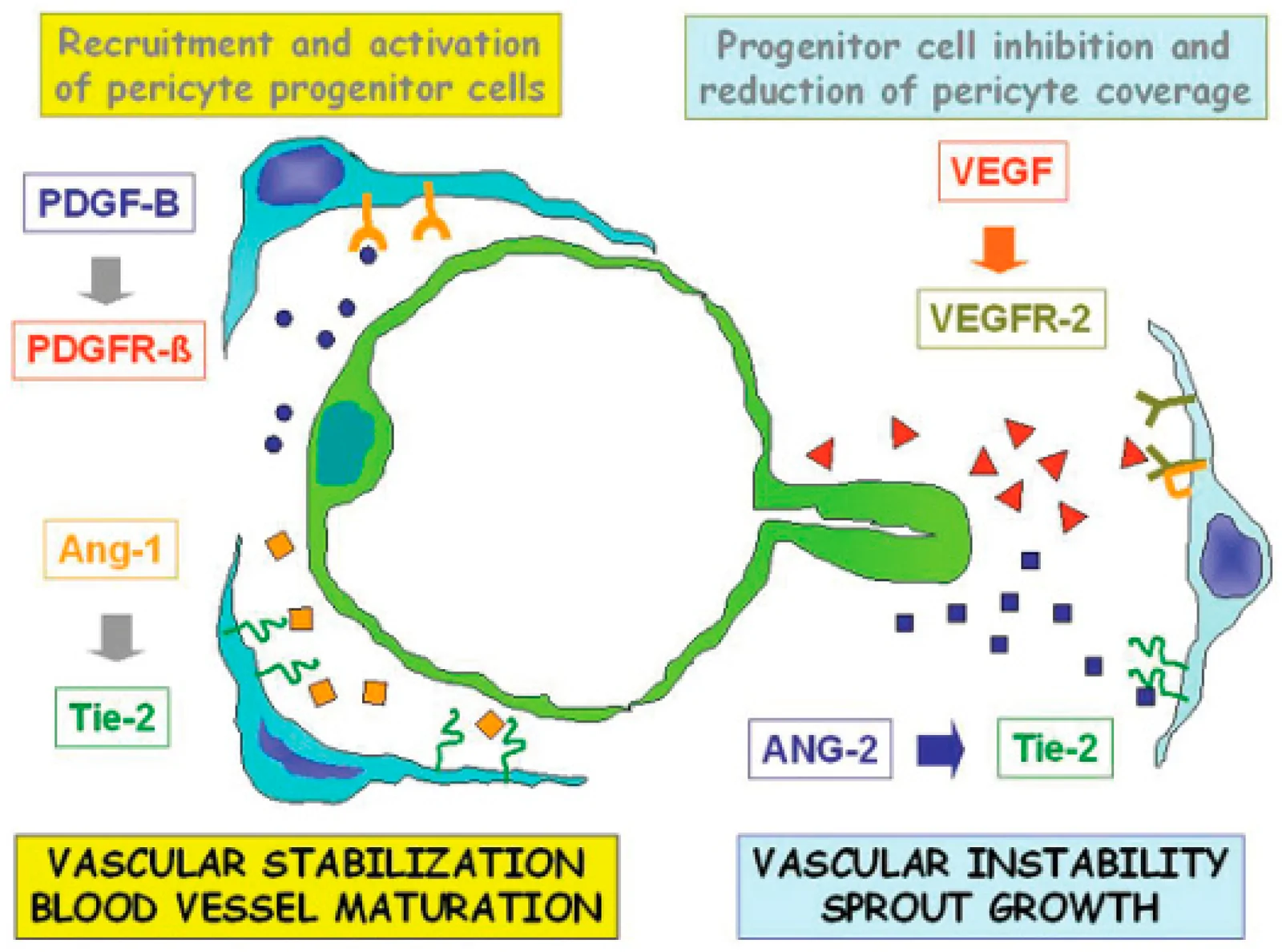
Paracrine PDGF and VEGF signaling axes controlling pericyte recruitment and sprout dynamics. Endothelial-derived PDGF-B stimulates pericyte precursor proliferation and recruitment via PDGFR-β activation, facilitating mural investment and vessel stabilization. Conversely, sprouting endothelial cells release VEGF, inducing VEGFR-2/PDGFR-β complex assembly that suppresses PDGFR-β signaling, destabilizes pericyte coverage, and promotes sprout regression. Reproduced from [12].

**Figure 2:**
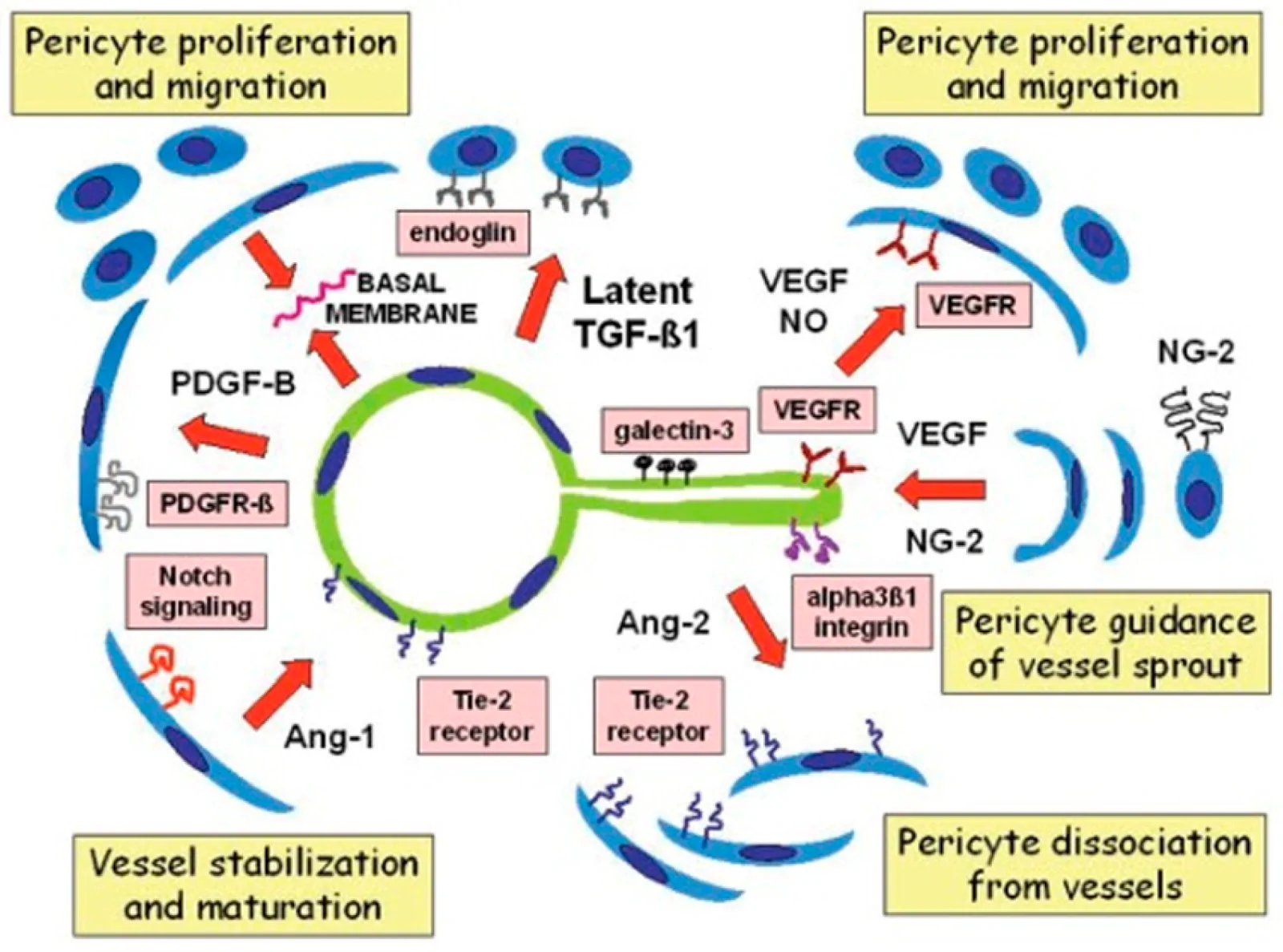
Molecular crosstalk regulating endothelial–pericyte dynamics during vessel sprouting and maturation. Sprouting vessel segments (**right**) undergo pericyte dissociation driven by Ang2/Tie2 axis activation. Pericytes guide sprout extension through paracrine VEGF release and soluble NG-2 deposition. Migrating endothelial cells further drives pericyte recruitment via VEGF and nitric oxide (NO) generation. Quiescent vessel stabilization (**left**) relies on tight pericyte coverage, PDGF-B/PDGFR-β engagement, Ang-1/Tie-2 activation, Notch signaling, and basement membrane deposition. Reproduced from [12].

Pericyte-derived Angiopoietin-1 (Ang1) engages endothelial Tie2 receptors to enforce microvascular maturation and structural stability [8]. Loss of Tie2 or Ang1 leads to embryonic lethality characterized by microvascular rupture, defective basement membrane assembly, and impaired pericyte attachment [9,10]. Conversely, Ang2 antagonizes Tie2 activation, promoting pericyte detachment and vascular destabilization [11] (Figure 1 and Figure 2).

Pericytes interact directly with endothelial cells through N-cadherin-mediated junctional complexes and focal contacts [13]. Elevated levels of prostaglandin E2 (PGE2) within the tumor microenvironment disrupt these intercellular junctions by downregulating N-cadherin via EP1 and EP4 receptor pathways [13].

General inclusion criteria of this article are limited to peer-review journal articles published in English. Exclusion criteria include studies that do not primarily focus on tumor pericytes or studies that do not provide enough details on the methodology or have incomplete results necessary for analysis. The database searched is PubMed; the search terms are angiogenesis, pericytes, and tumor progression. The search period is 1995–2026; the study selection procedure follows two screening steps: first by title and abstract, and then by full text.

## 2. Pericytes in Tumor Vessels

In malignant tissues, pericytes are primarily concentrated along active angiogenic fronts, where they exhibit striking structural and functional dysfunctions [14,15,16]. Pericyte detachment or structural abnormality directly compromises vascular barrier integrity, fueling tumor vessel hyperpermeability [17,18,19]. Endothelial PDGF-B production and pericytic PDGFR-β expression remain pivotal for mural cell recruitment to tumor vessels [20]. Under microenvironmental stress, vascular pericytes can undergo pericyte-to-fibroblast transition (PFT), detaching from the vessel wall and transdifferentiating into cancer-associated fibroblasts (CAFs) that actively accelerate tumor progression [21].

Pericytes participate in complex reciprocal crosstalk within the TME, influencing endothelial turnover, immune evasion, lineage transitions, tumor cell extravasation, epithelial-to-mesenchymal transition (EMT), and pre-metastatic niche establishment [22,23]. Classic EMT involves downregulation of epithelial adhesion molecules (such as E-cadherin and claudins) alongside upregulation of mesenchymal determinants (N-cadherin, vimentin, FSP1, and α-SMA) [24]. Alternatively, cancer cells undergoing pericyte-like transdifferentiation acquire mural cell properties, allowing them to co-opt the perivascular niche directly. While classical EMT equips tumor cells with migratory capabilities to invade the extracellular matrix and metastasize, pericyte-like transdifferentiation enables malignant cells to integrate into the vessel wall, regulating microvascular permeability and local blood flow under hypoxic conditions.

Classical EMT correlates with broad systemic dissemination, while pericyte-like transition directly contributes to structural vessel integrity, therapeutic resistance, and tumor survival under hypoxia. In this context, epithelial markers, including E-cadherin, decrease, while pericyte-like markers, including N-cadherin, α-SMA, and NG2 increase [25]. Targeting the PDGF signaling improves hypoxia-induced EMT of cancer cells and metastasis formation [26].

Single-cell transcriptomics and lineage-tracing studies confirm that pericytes exhibit broad diversity. Their molecular profiles and physiological roles are shaped significantly by their microenvironment and developmental origins [27].

Pericytes display heterogeneous behavior across different tumor types with variations in their structural density dictating distinct pathological states within the tumor microenvironment (TME) (Table 1), because they originate from diverse embryonic lineages, adapt dynamically to unique tissue microenvironments, and possess high cellular plasticity that alters their molecular marker expression, structural coverage, and paracrine signaling [28].

Rather than acting via a single mechanism, both increased and decreased pericyte coverage drive tumor progression through entirely different hemodynamic, metabolic, and cellular pathways.

Both increased and decreased pericyte coverage associate with tumor progression because they reflect two distinct pathological states of the tumor vasculature: excessive coverage stabilizes aggressive, well-perfused neo-vessels that fuel growth, whereas deficient coverage creates chaotic, leaky vessels that induce severe hypoxia and drive aggressive invasion and metastasis [26].

## 3. Pericytes and Normalization of Tumor Vasculature

Because tumor pericytes are highly plastic, they represent a major therapeutic target in oncology. The goal of pericyte-targeted therapy is to normalize tumor vasculature. The “normalization window” is a brief, temporary period after anti-angiogenic therapy when chaotic tumor blood vessels become more stable, structured, and efficient. This brief phase improves drug delivery and oxygen flow inside the tumor, helping treatments like chemotherapy and immunotherapy work better. Normalization of tumor vasculature is a therapeutic concept where abnormal, leaky, and chaotic blood vessels in a tumor are temporarily repaired to become more normal and structured. This process improves blood flow, increases oxygen levels, and helps chemotherapy and immune cells reach cancer more effectively. By restoring pericyte coverage, the chaotic and leaky tumor blood vessels are “normalized,” which decreases hypoxia, boosts drug delivery, and exposes the tumor to the immune system [29]. Normalization of the tumor microenvironment, particularly vascular and matrix normalization, fixes low oxygen levels, lowers fluid pressure, and turns on endothelial adhesion molecules, which lets helpful effector immune cells move easily into the core of the tumor [30].

Natural products focusing on the restoration of pericellular function and tumor vessel normalization can significantly improve the interaction between pericytes and endothelial cells. Traditional anti-angiogenic strategies focus primarily on vessel pruning, destroying existing blood vessels to starve the tumor. However, this intensive depletion often triggers severe intratumoral hypoxia, fueling cancer progression, metastasis, and therapeutic resistance. In contrast, vascular normalization aims to remodel pathologically disorganized, leaky vessels into a functionally and structurally mature network, thereby improving blood perfusion, relieving hypoxia, and enhancing the delivery of chemotherapeutic drugs and immune cells.

Using anti-angiogenic agents with immunotherapy enhances clinical outcomes by normalizing abnormal tumor blood vessels. This normalization decreases immunosuppression, boosts immune cell infiltration into the tumor microenvironment, and works synergistically with immune checkpoint inhibitors to extend progression-free and overall survival in cancers such as renal cells, hepatocellular, and non-small cell lung cancer [31]. Therapies target pericyte signaling pathways (like PDGF-BB and VEGF receptors) to promote lasting vascular normalization, supporting both targeted treatments and immunotherapies [32]. Tumor cells recruit pericytes to form new blood vessels; however, defective interactions with endothelial cells lead to chaotic, leaky vasculature that facilitates cancer cell entry into the bloodstream. Excessive depletion of pericytes paradoxically increases metastasis by over-pruning tumor blood vessels, resulting in severe intratumoral hypoxia. This hypoxia activates HIF-1α signaling, induces EMT, upregulates the Met receptor, and promotes aggressive invasion and dissemination of cancer cells [26]. Pericyte depletion toxicity includes vascular destabilization—causing vessel leakiness and abnormal microvasculature—and metastatic spread, as loss of pericytes can paradoxically enhance local intravasation, speeding up distant metastasis and tumor cell shedding.

## 4. Pericytes and Tumor-Associated Macrophages (TAMs)

Pericytes in the tumor microenvironment often promote an immune-desert or immunosuppressive state by recruiting M2-polarized macrophages and shielding cancer cells from T-cell responses [33]. TAMs can release angiogenic cytokines and preserve the pericyte-like phenotype of mesenchymal stem cells in the vascular environment [34]. When perivascular niches are forming, TAMs can attract pericytes [35]. Ablation of NG2 in myeloid cells decreases TAM recruitment and pericyte–endothelial cell interaction in an intracranial B16F10 melanoma model [36]. TAMs secrete factors like CECR1 (extracellular adenosine deaminase) that stimulate pericyte migration, helping stabilize chaotic new tumor blood vessels [37].

## 5. Pericyte and Pre-Metastatic Niche

Pericytes at the secondary sites can detach from vessels, undergo a phenotypic transformation, increase the synthesis of extracellular matrix components, create a pre-metastatic niche, and favor the invasion of hematogenous metastatic cancer cells. Vascular cell adhesion molecule-1 (VCAM-1), intercellular adhesion molecule-1 (ICAM-1), matrix metalloproteinases-2 and -9 (MMP-2 and MMP-9), which are secreted by pericytes and direct endothelial cell migration, are among the molecules engaged in extracellular matrix remodeling [38]. Pericytes in the pre-metastatic niche are altered, including reduced numbers and weakened or absent connections with endothelial cells and the basement membrane, resulting in the formation of highly permeable vessels [39]. Lugassy et al. [40] described the migration capabilities of melanoma cells along the outside of vessels, defined as “extravascular migratory metastasis”, taking a pericyte-like position.

## 6. Pericytes as Anti-Tumor Agents

The potential of pericytes as anti-tumor targets is supported by the synergistic benefits observed when combining anti-endothelial and anti-pericytic approaches [41,42]. VEGF inhibition eliminates tumor vessels without removing pericytes [14]. VEGF inhibitors, an antiangiogenic therapy that targets endothelial cells, cause tumor vasculature to recede and reduce tumor size [43]. This results in vascular normalization, which is marked by improved pericyte coverage, tumor perfusion, and chemotherapeutic sensitivity [29]. Since pericytes provide a framework for the quick regrowth of tumor vasculature, removal of VEGF inhibition results in tumor regrowth [44]. Stripping pericyte coverage leaves exposed, vulnerable endothelial structures, providing a mechanistic rationale for combined therapeutic strategies targeting both vascular compartments. Combined treatment or pre-treatment with anti-PDGF-B/PDGFBR-β reducing pericyte coverage increases the success of anti-VEGF treatment in the mouse RIP1-TAG2 model [45]. Extensive regression of endothelial cells was not observed in tumors after inhibition of PDGFR-β signaling [20]. STI571 (Gleevec, Imatinib), which targets PDGFRs and other receptor tyrosine kinases, did not reduce vascular density when given alone but did augment the effects of VEGF inhibitors [45]. In RIP1-TAG2 transgenic models and Lewis’s lung carcinomas treated with AG-013737 or VEGF-Trap, surviving pericytes either show tightened association with endothelial cells or complete dissociation from tumor vessels [46]. Treatment of RIP1-TAG2 tumors with anti-PDGFRβ antibody reduces pericytes, increases endothelial cell apoptosis but does not seem to reduce tumor vascular density [47]. In RIP1-TAG2 and xenotransplant cancers, the receptor tyrosine kinase inhibitor SU6668 detaches and decreases pericytes, limiting tumor growth [48,49]. It also inhibits PDGFR-β signaling. Imatinib causes tumor-associated PDGFRβ1 pericytes to undergo apoptosis, which disrupts the integrity of the tumor vasculature [50]. Lewis’s lung carcinomas showed a progressive decrease in pericytes after treatment with a novel selective PDGF-B blockade DNA aptamer AX102, which inhibits the function of PDGF-B [51]. In both zebrafish and murine models of angiogenesis, Murphy et al. [52] produced a series of selective type II inhibitors of PDGFR-β and B-RAF targets for pericyte recruitment and endothelial survival, respectively. They showed that dual inhibition of both PDGFR-β and B-RAF exerted synergistic anti-angiogenic activity. In human neuroblastoma-bearing mice, the anti-tumor efficacy of liposomal chemotherapy is increased by targeting pericytes and endothelial tumor cells with a combination of a peptide ligand of aminopeptidase A (APA), which was identified by phage display technology for delivery of liposomal doxorubicin (DXR) to perivascular tumor cells, and aminopeptidase N (APN)-targeted liposomal DXR [53]. Other studies with the aim to target pericytes have been conducted in experimental tumor models [54,55,56], and even in a human trial in advanced renal cell carcinoma [57]. In diverse solid malignancies, including glioma, Lewis lung carcinoma, and breast cancer, treatment with a dual-action antibody that concurrently activates Tie2 and inhibits Ang2, as well as restoring pericytic PDGFR-β expression while reinforcing endothelial tight junctions via VE-cadherin and claudin-5 upregulation.

## 7. Future Directions and Open Questions

Lack of tumor specificity, drug resistance mechanisms, and compensatory angiogenic pathways are tightly interconnected challenges associated with pericyte depletion. Lack of tumor specificity: pathways driving pericyte and endothelial recruitment (such as PDGF-BB/PDGFR-β and VEGF) are also utilized in wound healing and physiological tissue repair in healthy organs. Resistance mechanisms: depleting pericytes induces an inflammatory and hypoxic stress response that actively recruits protective bone marrow-derived cells and tumor-associated macrophages to shield the endothelium. Compensatory angiogenic pathways: blockade of primary survival signals prompts the upregulation of alternative pro-angiogenic cascades, such as FGFs, Ang2, and the EphrinB2–EphB4 axis.

Translating preclinical strategies that target tumor pericytes into clinical practice has largely stalled because pericytes exhibit complex functional plasticity, and laboratory models oversimplify human tumor microenvironments. Eradicating or stripping pericytes often worsens tumor hypoxia and accelerates metastatic spread rather than curing the cancer [26].

As concerns current controversies regarding pericyte targeting vs. vessel normalization strategies, the debate centers on whether to destroy tumor blood vessels by stripping away stabilizing pericytes or to coax them into a normal, mature state to improve drug and immune cell delivery. Pericyte targeting aims to starve tumors by destabilizing vessels, whereas vessel normalization seeks to optimize perfusion, reduce hypoxia, and boost immunotherapy efficacy [58].

Clinical translational evidence targeting tumor pericytes demonstrates a distinct hierarchical gap: robust proof of concept for pericyte modulation exists in animal models, whereas human clinical efficacy largely derives from anti-angiogenic multi-kinase inhibitors where benefits cannot be solely or directly attributed to pericyte regulation (Table 2).

The delicate balance between targeting tumor pericytes and preserving normal tissue vascular stability represents a critical frontier in modern oncology. The primary clinical challenge lies in safely disrupting the tumor-associated pericytes that fuel malignancy without sabotaging systemic vascular stability, healthy tissue repair, or driving treatment resistance.

Recent single-cell multi-omics and spatial transcriptomics have revealed that tumor pericytes are not a uniform population of vessel-stabilizing cells. Instead, high-resolution profiling shows profound phenotypic heterogeneity, uncovering specialized subsets (such as GPR116^+^ pericytes) that actively drive immune suppression, matrix remodeling, and metastasis rather than just passively supporting chaotic tumor blood vessels.

Existing experimental models to study tumor pericytes face major hurdles because they struggle to replicate the dynamic plasticity, high heterogeneity, and complex physical interactions of the real in vivo tumor microenvironment. Standard two-dimensional (2D) cultures completely lack spatial structure, while animal models often fail to mimic human-specific pericyte signaling [59]. 

## Figures and Tables

**Table 1:** **Comparative Analysis Across Tumor Types.** Pericyte density, attachment quality, and molecular markers vary depending on the primary tissue of origin and the dominant mode of vascularization within the tumor.

Tumor Type	Typical Pericyte PhenoType & Coverage Status	Primary Pathological Function
**Glioblastoma (GBM)**	**Increased Coverage** (Aberrant proliferation/hyper-density)	Glioma stem cells frequently transdifferentiate into pericytes to build extensive, atypical perivascular networks. This stabilizes microvessels against anti-angiogenic therapies and maintains a protective niche for cancer stem cells.
**Renal Cell Carcinoma (RCC)**	**Increased Coverage** (Highly invested mature microvessels)	RCC relies heavily on well-perfused, mature microvascular architecture to satisfy high metabolic requirements. These densely covered vessels act as a continuous fuel source for primary tumor growth.
**Breast & Pancreatic Carcinoma**	**Decreased Coverage** (Patchy, structurally detached pericytes)	Promotes an aggressive angiogenic switch, lose pericyte attachment leaves naked endothelial sprouts vulnerable to constant proliferation, compounding structural vessel chaos.
**Colorectal Carcinoma (CRC)**	**Highly Variable/Decreased** (Prone to local detachment)	Hematogenous extravasation of tumor cells into the systemic circulation is directly facilitated by a shift in vascular tone caused by disrupted pericyte–endothelial communication, which frequently involves regional downregulation of N-cadherin.

**Table 2:** **Hierarchical Framework of Translational Evidence.**

Level of Evidence	Primary Target/Mechanism Studied	Main Observational Endpoints	Translational Gap & Confounding Factors
**1. Animal Experiments (Preclinical Models)**	Selective PDGFRβ ablation, genetic knockouts, Diphtheria-toxin pericyte depletion, targeted ADCs	Tumor vessel destabilization, loss of mural coverage, vessel regression, acute hypoxia, modulation of metastasis	High selectivity achieved via genetic tools rarely translates to small-molecule clinical pharmacology; paradoxically, chronic depletion can trigger tumor hypoxia and metastasis.
**2. Early Clinical Studies (Phase I/II Monotherapy)**	Pericyte-directed targets (e.g., imatinib, olaratumab, anti-PDGFRβ mAbs, affibodies)	Safety, pharmacokinetics, reduction in intratumoral interstitial fluid pressure (IFP)	Minimal single-agent objective response rate (ORR) in non-oncogene-addicted solid tumors; pericyte marker modulation is inconsistent across biopsies.
**3. Multi-Targeted Anti-Angiogenic Regimens**	Dual VEGFR/PDGFR/KIT/FGFR TKIs (e.g., sunitinib, sorafenib, axitinib, regorafenib)	Progression-free survival (PFS), overall survival (OS), microvessel density reduction	Broad off-target inhibition of endothelial cells, tumor cell proliferation, and immune subsets obscures pericyte-specific contributions.

## Data Availability

No new data were created or analyzed in this study. Data sharing is not applicable to this article.
